# Phase 1 Randomized Controlled Trial of the Safety and Immunogenicity of the SARS-CoV-2 (Omicron BA.5) mRNA-CR-04 Vaccine in Adults 18–49 Years of Age

**DOI:** 10.1093/ofid/ofaf689

**Published:** 2025-11-26

**Authors:** Abdi Naficy, Mireille Venken, Yingmei Xi, Mark Loughrey, Giulietta Maruggi, Hema Sharma, Kunal Aggarwal, Daniel Brune, Bach-Yen Nguyen

**Affiliations:** Vaccines Clinical Sciences, GSK, Rockville, Maryland, USA; Vaccine Global Safety, GSK, Wavre, Belgium; Development Biostatistics, GSK, Belmont, Massachusetts, USA; Vaccine Global Safety, GSK, London, UK; Vaccine Discovery Technology, GSK, Cambridge, Massachusetts, USA; Vaccines Clinical Sciences, GSK, London, UK; Technical Research & Development, GSK, Rockville, Maryland, USA; Accelerated Enrollment Solutions, Peoria, Illinois, USA; Vaccines Clinical Sciences, GSK, Rockville, Maryland, USA

**Keywords:** COVID-19, immunogenicity, mRNA, safety, vaccine

## Abstract

**Background:**

This study (NCT05972993) evaluated a novel mRNA vaccine construct using the SARS-CoV-2 BA.5 Spike (S) protein as the model antigen (mRNA-CR-04).

**Methods:**

This first-in-human Phase 1, randomized, placebo-controlled trial enrolled 72 participants in Part A (sentinel vaccination and dose escalation) and 42 in Part B (dose exploration). Adult participants 18–49 years of age were randomized in 3 groups to receive one dose of mRNA-CR-04 (either 10, 30, or 100 µg) or placebo (3:1) in Part A, and 3 µg, 10 µg, or placebo (3:3:1) in Part B. Vaccine safety and immunogenicity in terms of neutralizing titers were assessed until 6 months postinvestigational product administration.

**Results:**

Solicited adverse events (AEs) were mostly mild to moderate and transient. In Part A, Grade 3 reactogenicity was only observed in the 100 µg group (*n* = 3, 16.7%), and Grade 3 nonsolicited AEs only occurred as causally unrelated serious AEs in 2 participants. No safety concerns deemed causally related to mRNA-CR-04 were raised on review of clinical safety data and clinical laboratory test results. All doses elicited notable neutralizing titers against the vaccine-encoded SARS-CoV-2 BA.5 variant and induced cross-neutralizing titers against the wild type (D614G) variant. The magnitude of the immune response tended to increase with dose. Neutralizing titers waned by Month 6 but remained above baseline levels.

**Conclusions:**

The investigational mRNA-CR-04 vaccine was generally well tolerated, and all doses induced a robust immune response against the encoded antigen at doses ranging between 3 and 100 µg. Further investigation of potential vaccine candidates using this novel mRNA platform is warranted.

## Teaser

As anticipated, the investigational mRNA-CR-04 vaccine elicited robust immune responses against the encoded antigen in adults that persisted for at least 6 months. No safety concerns deemed causally related to mRNA-CR-04 were observed for doses between 3 and100 µg.

Vaccines based on mRNA technology were the subject of intensive research for several decades prior to the severe acute respiratory syndrome coronavirus 2 (SARS-CoV-2) pandemic when their efficacy and safety were validated [1]. mRNA coronavirus disease 2019 (COVID-19) vaccines have been administered to billions of the world's population and are estimated to have prevented millions of deaths due to COVID-19 [2–4]. mRNA vaccines continue to be the cornerstone of ongoing COVID-19 control in many countries. Their success has seen the acceleration of mRNA vaccine development targeting a range of respiratory viruses [5].

mRNA vaccines are comprised of synthetic mRNA strands modified to express one or more specific antigens formulated within lipid nanoparticles to deliver the mRNA to the cell cytoplasm where translation occurs [5, 6]. Because mRNA is delivered in the cell cytoplasm where proteins are expressed, there is no risk of insertion mutations occurring in the host nucleus. The lipid nanoparticles act as strong adjuvants, leading to robust humoral and cellular immune responses, extending their potential utility to older adults in whom effective adjuvants may be needed to overcome immunosenescence [6]. The type of modifications made to the RNA molecule, the mRNA dose, and the characteristics of the lipid nanoparticles can impact biodistribution, translation efficiency, the duration of antigen production, and the innate immune response to vaccination [6, 7].

mRNA vaccines can be manufactured relatively quickly, cost-effectively, and at large scale [8]. The mRNA sequence can be easily modified to target emerging pathogenic strains of bacteria or viruses, allowing rapid and flexible responses to mutating pathogens. Multiple mRNA molecules encoding for distinct antigens targeting the same pathogen or multiple pathogenic variants can be codelivered within the same vaccine. Clinically, this approach has been harnessed in bivalent SARS-CoV-2 booster vaccine formulations, such as those incorporating mRNAs for both the ancestral spike protein and emerging variants like BA.4/BA.5 [9]. Multivalent mRNAs aim to broaden immune protection against currently circulating strains, or to increase the number of antigenic epitopes available for immune stimulation, as currently being investigated in the development of cytomegalovirus and influenza mRNA-based vaccines [10, 11]. These features have been leveraged since the end of the SARS-CoV-2 pandemic, with manufacturers now producing variant-specific, multicomponent COVID-19 booster vaccines at regular intervals as the SARS-CoV-2 virus continues to evolve [5, 12], in a similar iteration of the process used for production of seasonal influenza vaccines [13].

This study was conducted to evaluate a novel mRNA vaccine construct using the SARS-CoV-2 BA.5 Spike (S) protein as the model antigen (mRNA-CR-04 vaccine).

This first-in-human Phase 1 dose escalation and dose exploration trial was conducted to assess the safety and immunogenicity of the investigational mRNA-CR-04 vaccine administered as a booster dose in healthy or medically stable adults previously vaccinated with an mRNA COVID-19 vaccine.

## METHODS

### Study Design

**Figure 1.**
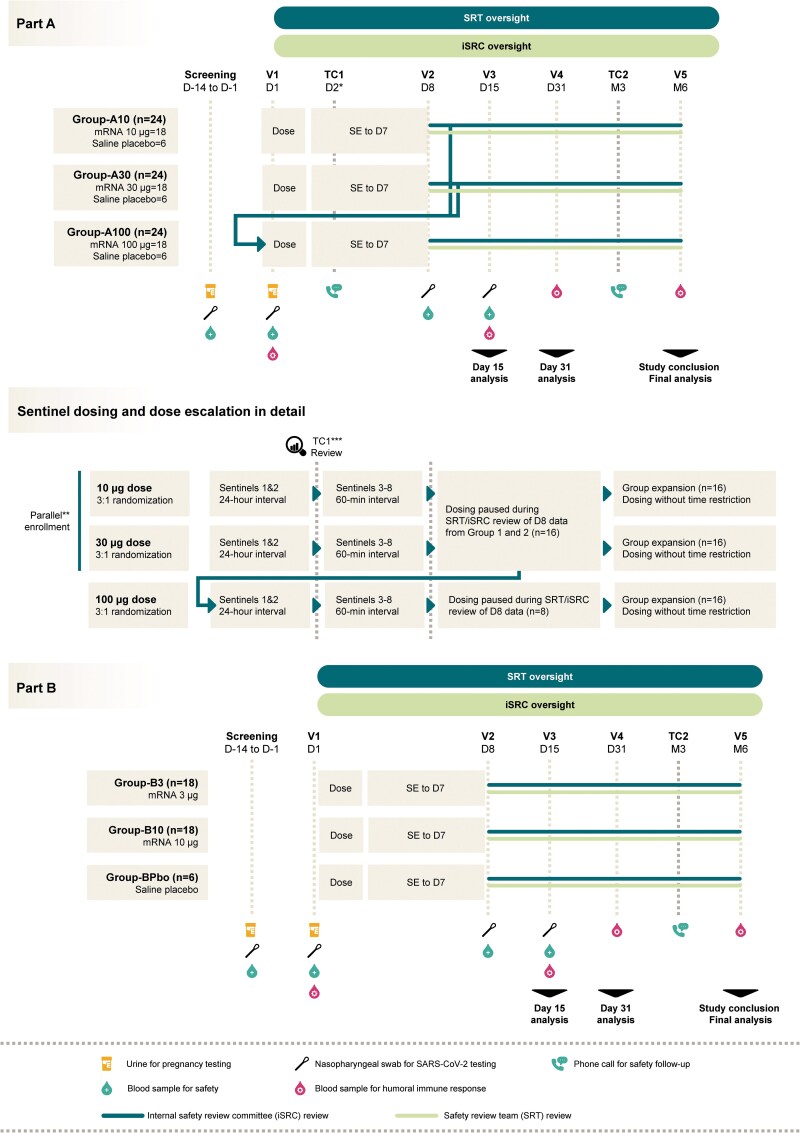
Study design. D: Day; iSRC: internal safety review committee; M: Month; mRNA: mRNA-CR-04 vaccine; n: number of participants; SARS-CoV-2: severe acute respiratory syndrome coronavirus 2; SE: Solicited events; SRT: safety review team; TC: telephone call; V: Visit. * TC1 D2 only for sentinel participants in each group. ** The first participant enrolled in Group 1. *** TC1 review by the SRT on blinded data.

This Phase 1, randomized, observer-blind, placebo-controlled trial was conducted at 4 centers in the United States (US) (NCT05972993, clinical trial report date 24 January 2025). In the dose escalation phase (Part A), 24 participants in each of 3 groups (Groups 1, 2, and 3) were randomized in a 3:1 ratio to receive either 10, 30, or 100 µg of mRNA-CR-04 (vaccine recipients in the 3 groups are referred to here as Group-A10, Group-A30, and Group-A100) or placebo (placebo recipients in the 3 groups are combined and referred to here as Group-APbo) (Figure 1). Eight sentinel participants (6 receiving mRNA-CR-04 and 2 receiving placebo) were enrolled first in each group. Enrollment was staggered such that the first 2 sentinel participants in Groups 1 and 2 were dosed and followed up for 24 hours before dosing the remaining sentinel participants. Unblinded safety data until Day 8 were reviewed by an internal safety review committee to confirm that dosing of nonsentinel participants could begin, and that sentinel participants in Group 3 could continue. All sentinel participants were observed for at least 60 minutes before dosing of the next participant took place.

After completion of the prespecified interim analysis of Day 15 data from all participants in Part A, the notable immune responses in the 10 µg vaccine recipients prompted the addition of Part B to the study to explore an even lower vaccine dose. Forty-two participants in Part B were randomized in a 3:3:1 ratio to receive one dose of either mRNA-CR-04 3 µg (Group-B3), mRNA-CR-04 10 µg (Group-B10), or saline placebo (Group-BPbo). Safety and immunogenicity were assessed until 6 months after the administration of investigational product (IP) in all study participants.

### Study Objectives

The primary study objective was to evaluate the safety and reactogenicity of mRNA-CR-04 until Day 31 postvaccination. Secondary objectives were to assess vaccine safety throughout the entire study until Month 6, and to evaluate neutralizing titer responses against the vaccine-encoded SARS-CoV-2 BA.5 variant, and cross-neutralization against the wild type (WT) SARS-CoV-2 (D614G) variant up to study conclusion.

### Ethics Approval and Study Oversight

The study was conducted in accordance with Good Clinical Practice guidelines and applicable laws and regulations. The protocol was independently reviewed and approved by the central Institutional Review Board (IRB), Advarra, IRB organization number 0000635 and registration number 00000971. All participants gave written informed consent prior to enrollment.

Safety monitoring was performed by an internal safety review team which conducted blinded data review until the Part A Day 15 interim analysis, and unblinded data review subsequently, and a distinct internal safety review committee consisting of no individuals involved with the study conduct (unblinded data review). Safety holding rules were specified in the protocol and are provided in the Supplementary material.

### Study Participants

Participants were healthy or medically stable adults 18–49 years of age who had completed a 2-dose primary series and one or more booster doses with mRNA COVID-19 vaccines (only Moderna or Pfizer vaccines) at least 6 months prior to screening. Participants were required to be negative for SARS-CoV-2 infection by real-time polymerase chain reaction test during screening. Individuals with a history of, or risk factors for, pericarditis or myocarditis were excluded. The full list of eligibility criteria is provided in the Supplementary material.

### Randomization and Blinding

A web-based randomization was used and based on a permutation block algorithm stratified by study part and dose escalation step. Part A was observer-blind to participant, site, and sponsor personnel involved in the clinical evaluation. Study participants and site personnel involved in the clinical evaluation of the participants remained blinded during Part B.

### Study Vaccine and Placebo

The mRNA-CR-04 vaccine was developed using sequence-optimized mRNA that is capped, polyadenylated, and incorporates modified nucleosides. The mRNA encodes the full-length Spike (S) protein of the SARS-CoV-2 Omicron BA.5 variant (GSAID ID: EPI_ISL_12268495) and includes the S-2P prefusion stabilizing mutation [14]. To enhance protein expression in human cells, the mRNA was further optimized using a proprietary algorithm for efficient protein expression in human cells. Key features of the mRNA construct include optimized noncoding regions (proprietary 5′ and 3′ untranslated regions) and a poly-A tail. The mRNA was encapsulated in lipid nanoparticles (composed of a cationic lipid, PEGylated lipid, sterol lipid, and phospholipid) with >90% encapsulation efficiency. The placebo consists of normal saline (sodium chloride 0.9%). The injected volume was 0.3 mL.

### Safety Evaluation

Adverse events (AEs) were solicited using electronic diaries for 7 days post-IP administration. The grading scale for AE intensity is provided in Supplementary Table 1. Unsolicited AEs were recorded for 30 days post-IP administration and serious adverse events (SAEs), adverse events of special interest (AESI), medically attended AEs, and pregnancies were captured during the entire study. The protocol-defined AESI were myocarditis and pericarditis, virologically confirmed COVID-19, anaphylaxis or severe hypersensitivity within 24 hours after IP administration, and the onset of potential immune-mediated disorders. Electrocardiographs (ECGs) and serum cardiac troponin I levels were measured at screening and Day 8. Hematology and serum chemistry were assessed at screening and Days 1, 8, and 15 (Figure 1).

### Immunogenicity Evaluation

Anti-SARS-CoV-2 neutralizing titers against BA.5 and WT (D614G) variants were measured from serum samples collected at baseline, Day 15, Day 31, and Month 6 using a pseudo-neutralization assay at Nexelis laboratory (lower limit of quantitation [LLOQ] = 10) [15]. Serology testing for antibody to the SARS-CoV-2 nucleocapsid (N) protein was performed on Days 1, 15, and 31, and Month 6 to monitor for undetected SARS-CoV-2 infections using an anti-SARS-CoV-2 immunoassay (Roche Elecsys) for the in vitro qualitative detection of antibodies to N protein in human serum. The assay uses a recombinant protein representing the N antigen in a double-antigen sandwich assay format, which favors detection of high affinity antibodies against SARS-CoV-2 N antigen. The results are determined by comparing the electrochemiluminescence signal obtained from the reaction product of the sample with the signal of a cutoff value using the Roche COBAS 8000 (E602) instrument. Tests were conducted by Pharmaceutical Product Development.

### Statistical Analysis

In this early phase trial, all analyses were purely descriptive, and no hypotheses were statistically tested. Safety was evaluated on the exposed set, comprising all participants who received the study vaccine or placebo. Analysis of solicited AEs used the solicited safety set that included all participants who completed the electronic diary for solicited AEs. Immunogenicity was assessed using the per-protocol set for each time point, which was comprised of participants who received their assigned IP as per-protocol, were without major protocol deviations, were without intercurrent conditions or medications/vaccinations that may have interfered with immunogenicity, and had predose and postdose neutralizing titers against pseudovirus-bearing BA.5 S protein data for the relevant time point.

Placebo participants in the 3 Part A groups were pooled for the analysis of safety and immunogenicity as Group-APbo. Descriptive statistics (mean, median, standard deviation, range) were calculated for continuous variables, and categorical variables were computed using frequencies (number/percentage). The frequency of AEs was reported as percentages with 95% confidence intervals (CIs).

Neutralizing titers were described in terms of geometric mean titers (GMTs) and geometric mean ratios (GMRs) from baseline. GMTs were calculated by taking the inverse logarithm of the mean of the log titer transformations. Values below the LLOQ were given a value of LLOQ/2.

At each post-IP administration time point, the adjusted GMTs with their 95% CI were obtained using an analysis of covariance model on log_10_ transformed titers with treatment group as a fixed factor, and the log_10_ transformed Day 1 baseline value as a covariable. The number and percentage of participants (with exact 95% CI) with at least a 2-, 4-, and 8-fold increase in titer from Day 1 were calculated.

### Sample Size

With 18 participants receiving mRNA-CR-04 in each group, there was a 60.3% probability of observing at least one AE if the incidence rate was 5%, and 85% probability of observing at least one AE if the incidence rate was 10%.

## RESULTS

The study was conducted between 7 August 2023 and 14 October 2024.

### Part A

Among 107 individuals screened, 72 were enrolled and received their assigned IP in Part A and 69 (95.8%) completed the study (Supplementary Figure 1). Demographic and baseline characteristics of study groups are presented in Table 1. The median age of all participants at screening was 38.5 years (range 19–49), 59.7% (*n* = 43) were female, 75.0% (*n* = 54) were White, and 13.9% identified as Hispanic/Latino. A total of 73.6% (*n* = 53) of participants were N protein–antibody positive at Day 1.

**Table 1.** Demographic Characteristics of Participants in Parts A and B (Exposed Set)

Part A	Group-A10 *N* = 18		Group-A30 *N* = 18		Group-A100 *N* = 18		Group-APbo *N* = 18		Total *N* = 72	
	*n*	%	*n*	%	*n*	%	*n*	%	*N*	%
Age (y) at screening										
Median (range)	42.5 (19–49)		40.5 (24–49)		34.5 (24–49)		35.0 (28–49)		38.5 (19–49)	
Sex										
Male	8	44.4	8	44.4	6	33.3	7	38.9	29	40.3
Female	10	55.6	10	55.6	12	66.7	11	61.1	43	59.7
Ethnicity										
Hispanic or Latino	4	22.2	1	5.6	2	11.1	3	16.7	10	13.9
Not Hispanic or Latino	14	77.8	17	94.4	16	88.9	15	83.3	62	86.1
Race										
Asian	1	5.6	2	11.1	0	0	0	0	3	4.2
Black or African American	1	5.6	4	22.2	3	16.7	2	11.1	10	13.9
Native Hawaiian or Other Pacific Islander	1	5.6	0	0	0	0	0	0	1	1.4
White	14	77.8	12	66.7	14	77.8	14	77.8	54	75.0
Not reported	1	5.6	0	0	1	5.6	2	11.1	4	5.6
N* protein										
Positive	12	66.7	12	66.7	15	83.3	14	77.8	53	73.6
Negative	6	33.3	6	33.3	3	16.7	4	22.2	19	26.4

Part B	Group-B3 *N* = 18		Group-B10 *N* = 18		Group-BPbo *N* = 6		Total *N* = 42	
	*n*	%	*N*	%	*n*	%	*n*	%
Age (y) at screening								
Median (range)	34.0 (19–49)		38.5 (22–49)		44.0 (30–49)		38.5 (19–49)	
Sex								
Male	10	56	8	44	3	50	21	50
Female	8	44	10	56	3	50	21	50
Ethnicity								
Hispanic or Latino	4	22	2	11	3	50	9	21
Not Hispanic or Latino	14	78	16	89	3	50	33	79
Race								
Black or African American	1	6	0	0	0	0	1	2
White	16	89	18	100	5	83	39	93
Not reported	1	6	0	0	1	17	2	5
N* protein								
Positive	18	100	18	100	6	100	42	100

*N*, number of participants; *n* (%), number (percentage) of participants in a given category; N*, nucleocapsid.

Part A: sentinel vaccination and dose escalation; Part B: dose exploration.

### Safety

**Figure 2.**
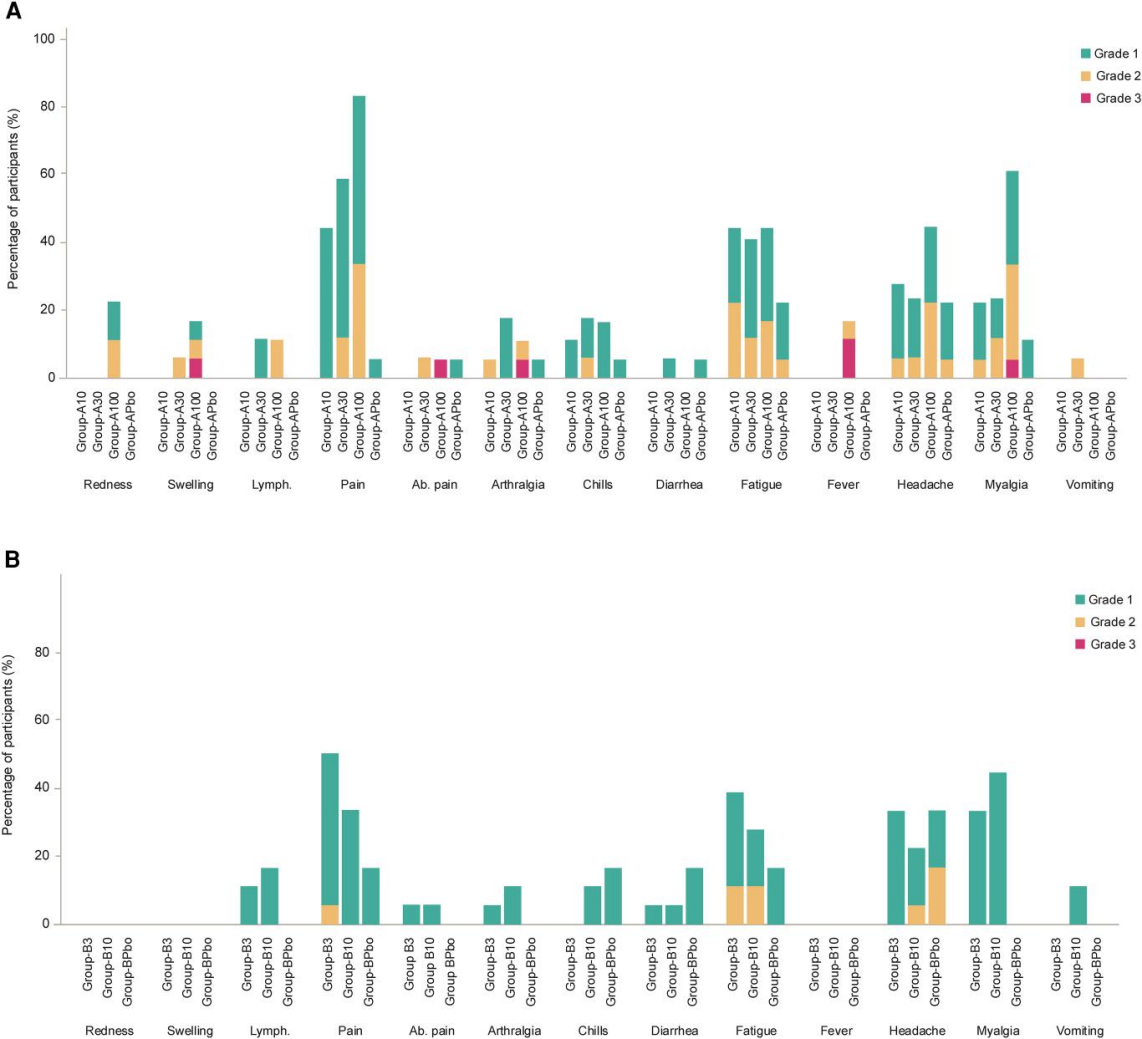
Solicited adverse events by dose group and maximum grade: (*A*) Part A, (*B*) Part B. Ab.: abdominal; Lymph: ipsilateral axillary lymphadenopathy Intensity scales for solicited events are provided in the Supplement.

The frequency and intensity of solicited local and systemic AEs appeared to be dose-related, with the highest percentage of participants reporting any solicited AE in Group-A100 (88.9%) versus 55.6% in Group-A10, 70.6% in Group-A30, and 38.9% in Group-APbo (Table 2). The most frequently reported solicited AEs were injection site pain (44.4% in Group-A10, 58.8% in Group-A30, 83.3% in Group-A100, vs 5.6% in Group-APbo), fatigue (44.4%, 41.2%, 44.4%, vs 22.2% in the respective groups), headache (27.8%, 23.5%, 44.4%, vs 22.2%), and myalgia (22.2%, 23.5%, 61.1%, vs 11.1%, respectively) (Figure 2*A*). Grade 3 solicited AEs were only reported in Group-A100 (*n* = 3, 16.7%): one participant with Grade 3 swelling, one with Grade 3 abdominal pain, arthralgia, fever, and myalgia, and one with Grade 3 fever. The median duration of solicited AEs was 1–2 days for a majority of AEs, and those with durations of 4 days or more were headache (*n* = 1, Group-APbo, 4 days), abdominal pain (*n* = 1, Group-A30, 4 days), and arthralgia (*n* = 1, Group-A10, 7 days in a participant with a history of osteoarthritis and anterior cruciate ligament tear and reconstruction).

**Table 2.** Safety Summary (Solicited Safety Set^a^ and Exposed Set)

Part A	Group-A10 *N* = 18		Group-A30 *N* = 18 (17^a^)		Group-A100 *N* = 18		Group-APbo *N* = 18	
	*n*	%	*n*	%	*n*	%	*n*	%
Any solicited AE (Day 1–7)	10	55.6	12	70.6	16	88.9	7	38.9
Local	8	44.4	11	64.7	16	88.9	1	5.6
Systemic	10	55.6	10	58.8	14	77.8	7	38.9
Any Grade 3	0	0	0	0	3	16.7	0	0
Local	0	0	0	0	1	5.6	0	0
Systemic	0	0	0	0	2	11.1	0	0
Any unsolicited AE (Day 1–30)	6	33.3	5	27.8	4	22.2	3	16.7
Grade 3	0	0	1	5.6	0	0	0	0
Related	3	16.7	1	5.6	0	0	1	5.6
Grade 3 related	0	0	0	0	0	0	0	0
Any MAAE until study end	3	16.7	2	11.1	3	16.7	1	5.6
Grade 3	1	5.6	1	5.6	1	5.6	0	0
Related	0	0	0	0	0	0	0	0
Any AESI until study end	0	0	0	0	1	5.6	1	5.6
Grade 3	0	0	0	0	0	0	0	0
Any SAE until study end	1	5.6	1	5.6	0	0	0	0
Related	0	0	0	0	0	0	0	0

Part B	Group-B3 *N* = 18		Group-B10 *N* = 18		Group-BPbo *N* = 6	
	*n*	%	*n*	%	*n*	%
Any solicited AE (Day 1–7)	14	77.8	10	55.6	3	50.0
Local	10	55.6	6	33.3	1	16.7
Systemic	11	61.1	9	50.0	2	33.3
Any Grade 3	0	0	0	0	0	0
Any unsolicited AE (Day 1–30)	8	44.4	2	11.1	1	16.7
Grade 3	0	0	0	0	0	0
Related	5	27.8	1	5.6	0	0
Grade 3 related	0	0	0	0	0	0
Any MAAE until study end	3	16.7	0	0	0	0
Grade 3	0	0	0	0	0	0
Related	0	0	0	0	0	0
Any AESI until study end	0	0	0	0	0	0
Any SAE until study end	0	0	0	0	0	0

Abbreviations: AE, adverse event; AESI, adverse event of special interest; MAAE, medically attended adverse event; *N*, number of participants; *n* (%), number (percentage) of participants in a given category; SAE, serious adverse event.

^a^The solicited safety set was used for the analysis of solicited local and systemic AEs. One participant (Group-A30) who did not complete the e-diary and who was lost to follow-up after Visit 1 was excluded from the solicited safety set.

Part A: sentinel vaccination and dose escalation; Part B: dose exploration.

The percentage of participants reporting any unsolicited AE was similar in each group (33.3% in Group-A10, 27.8% in Group-A30, 22.2% in Group-A100, vs 16.7% in Group-APbo) (Table 2). One was of Grade 3 intensity (Group-A30, attempted suicide). Unsolicited AEs considered by the investigator to be causally related to the IP were toothache and restless leg syndrome (*n* = 1, Group-A10), headache and nausea (*n* = 1, Group-A10), prothrombin time prolonged (*n* = 1, Group-A10), neutrophil count decreased (*n* = 1, Group-A30), and hyperhidrosis (*n* = 1, Group-APbo); all were Grade 1 in intensity and resolved.

Medically attended AEs until study end were reported by no more than 3 participants (16.7%) in each group, and none were considered causally related to the IP (Table 2). Two participants (one in Group-APbo and one in Group-A100) reported an AESI; both were cases of Grade 1 COVID-19 considered causally unrelated to the IP. Two participants reported 2 SAEs each: otitis media and otitis externa by one, suicide attempt and ankle fracture by the other, and none were considered causally related to the IP by the investigator. There were no deaths, and no participant discontinued the study due to an AE. No safety signals were observed from the clinical safety laboratory test results or review of ECGs (not shown).

### Immunogenicity

**Figure 3.**
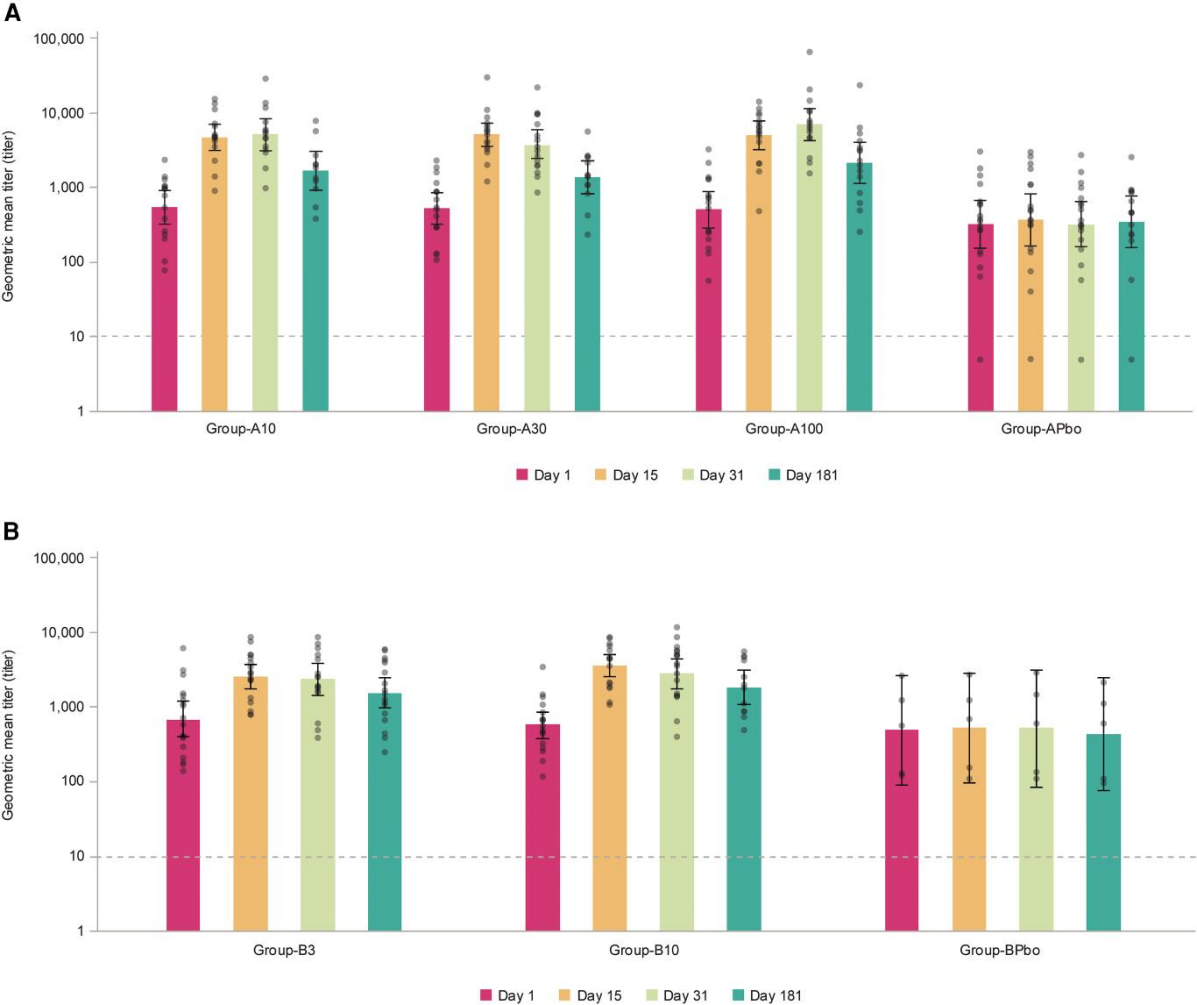
Unadjusted geometric mean titers for neutralizing titers against SARS-CoV-2 BA.5 by time point: (*A*) Part A, (*B*) Part B (per-protocol set). SARS-CoV-2: severe acute respiratory syndrome coronavirus 2 Circles represent individual datapoints, horizontal line represents the assay lower limit of quantitation, whiskers indicate 95% confidence intervals.

The composition of the per-protocol sets is provided in the Supplement (Supplementary Figure 1A). Between one and 4 participants were excluded from the Day 15 and Day 31 analyses, and between 3 and 7 from the Month 6 analysis, mainly due to missing endpoints or protocol deviations (Supplementary Figure 1A). Overall, 73.6% of participants had evidence of prior SARS-CoV-2 infection, and levels of neutralizing titers against SARS-CoV-2 BA.5 prior to IP administration were high (Figure 3*A*). Despite this, all but 3 participants (all in Group-A100) who received mRNA-CR-04 had at least a doubling in neutralizing titers against the SARS-CoV-2 BA.5 variant at Day 15, and at least 68.8% had a ≥ 4-fold change (Table 3). At Day 31, the percentage of participants with a ≥ 4-fold change in neutralizing titers from baseline levels was 85.7% in Group-A10, 70.6% in Group-A30, and 93.8% in Group-A100, versus 0% in Group-APbo. At Month 6, 63.6% of participants in Group-A10, 53.8% in Group-A30, and 66.7% in Group-A100 continued to have neutralizing titers at least 2-fold higher than at baseline.

**Table 3.** Fold-change From Baseline in Unadjusted SARS-CoV-2 BA.5 Neutralizing Titers (per-Protocol Set)

Time Point	Fold Change	*N*	*n*	% (95% CI)	*N*	*n*	% (95% CI)	*N*	*n*	% (95% CI)	*N*	*n*	% (95% CI)
**Part A**													
				Group-A10			Group-A30			Group-A100			Group-APbo
Day 15	<2	16	0	0.0 (0.0–20.6)	17	0	0.0 (0.0–19.5)	17	3	17.6 (3.8–43.4)	18	16	88.9 (65.3–98.6)
	≥2	16	16	100 (79.4–100)	17	17	100 (80.5–100)	17	14	82.4 (56.6–96.2)	18	2	11.1 (1.4–34.7)
	≥4	16	11	68.8 (41.3–89)	17	13	76.5 (50.1–93.2)	17	14	82.4 (56.6–96.2)	18	2	11.1 (1.4–34.7)
	≥8	16	8	50 (24.7–75.3)	17	8	47.1 (23.0–72.2)	17	12	70.6 (44.0–89.7)	18	1	5.6 (.1–27.3)
Day 31	<2	14	0	0.0 (0.0–23.2)	17	2	11.8 (1.5–36.4)	16	1	6.3 (0.2–30.2)	18	18	100 (81.5–100)
	≥2	14	14	100 (76.8–100)	17	15	88.2 (63.6–98.5)	16	15	93.8 (69.8–99.8)	18	0	0.0 (.0–18.5)
	≥4	14	12	85.7 (57.2–98.2)	17	12	70.6 (44.0–89.7)	16	15	93.8 (69.8–99.8)	18	0	0.0 (0.0–18.5)
	≥8	14	5	35.7 (12.8–64.9)	17	6	35.3 (14.2–61.7)	16	12	75.0 (47.6–92.7)	18	0	0.0 (0.0–18.5)
Month 6	<2	11	4	36.4 (10.9–69.2)	13	6	46.2 (19.2–74.9)	15	5	33.3 (11.8–61.6)	15	12	80.0 (51.9–95.7)
	≥2	11	7	63.6 (30.8–89.1)	13	7	53.8 (25.1–80.8)	15	10	66.7 (38.4–88.2)	15	3	20.0 (4.3–48.1)
	≥4	11	3	27.3 (6.0–61)	13	3	23.1 (5.0–53.8)	15	7	46.7 (21.3–73.4)	15	1	6.7 (0.2–31.9)
	≥8	11	1	9.1 (0.2–41.3)	13	3	23.1 (5.0–53.8)	15	4	26.7 (7.8–55.1)	15	1	6.7 (0.2–31.9)
**Part B**													
				Group-B3			Group-B10			Group-BPbo			
Day 15	<2	18	5	27.8 (9.7–53.5)	17	2	11.8 (1.5–36.4)	5	5	100 (47.8–100)			
	≥2	18	13	72.2 (46.5–90.3)	17	15	88.2 (63.6–98.5)	5	0	0.0 (0.0–52.2)			
	≥4	18	7	38.9 (17.3–64.3)	17	13	76.5 (50.1–93.2)	5	0	0.0 (0.0–52.2)			
	≥8	18	3	16.7 (3.6–41.4)	17	4	23.5 (6.8–49.9)	5	0	0.0 (0.0–52.2)			
Day 31	<2	17	5	29.4 (10.3–56.0)	17	4	23.5 (6.8–49.9)	5	5	100 (47.8–100)			
	≥2	17	12	70.6 (44–89.7)	17	13	76.5 (50.1–93.2)	5	0	0.0 (0.0–52.2)			
	≥4	17	5	29.4 (10.3–56.0)	17	11	64.7 (38.3–85.8)	5	0	0.0 (0.0–52.2)			
	≥8	17	3	17.6 (3.8–43.4)	17	3	17.6 (3.8–43.4)	5	0	0.0 (0.0–52.2)			
Month 6	<2	18	10	55.6 (30.8–78.5)	12	5	41.7 (15.2–72.3)	5	5	100 (47.8–100)			
	≥2	18	8	44.4 (21.5–69.2)	12	7	58.3 (27.7–84.8)	5	0	0.0 (0.0–52.2)			
	≥4	18	4	22.2 (6.4–47.6)	12	3	25.0 (5.5–57.2)	5	0	0.0 (0.0–52.2)			
	≥8	18	3	16.7 (3.6–41.4)	12	1	8.3 (.2–38.5)	5	0	0.0 (0.0–52.2)			

Abbreviations: CI, confidence interval; *N*, number with pre and corresponding postresults available; *n* (%), number (percentage) with the specified fold change; SARS-CoV-2, severe acute respiratory syndrome coronavirus 2.

All doses of mRNA-CR-04 elicited neutralizing titers against SARS-CoV-2 BA.5 (Table 4). There was a consistent trend toward a dose-response, notwithstanding the robust responses observed at the lowest 10 μg dose level. At Day 31, the SARS-CoV-2 BA.5 neutralizing titer GMR from baseline was 8.57 in Group-A10, 7.37 in Group-A30, and 13.46 in Group-A100. These neutralizing titers had waned by Month 6 but remained above baseline titers in all mRNA-CR-04 groups.

**Table 4.** Adjusted SARS-CoV-2 BA.5 Neutralizing Geometric Mean Titers and Geometric Mean Ratios From Baseline (per-Protocol Set)

Time Point	Group-A10			Group-A30			Group-A100			Group-APbo		
	*N*	GMT (95% CI)	GMR (95% CI)	*N*	GMT (95% CI)	GMR (95% CI)	*N*	GMT (95% CI)	GMR (95% CI)	*N*	GMT (95% CI)	GMR (95% CI)
**Part A**												
Day 1	16	550.72 (325.81–930.89)	-	17	526.95 (325.54–852.97)	-	17	504.01 (287.71–882.94)	-	18	320.87 (154.81–665.05)	-
Day 15	16	4271.90 (2751.76–6631.81)	9.25 (5.96–14.36)	17	4720.61 (3082.55–7229.14)	10.22 (6.68–15.66)	17	4795.20 (3132.57–7340.29)	10.38 (6.78–15.90)	18	453.93 (298.47–690.36)	0.98 (.65–1.50)
Day 31	14	4197.66 (2747.44–6413.36)	8.57 (5.61–13.10)	17	3607.13 (2463.89–5280.84)	7.37 (5.03–10.78)	16	6591.72 (4448.81–9766.85)	13.46 (9.08–19.94)	18	420.37 (288.08–613.40)	0.86 (.59–1.25)
Month 6	11	1414.22 (786.90–2541.66)	2.76 (1.53–4.95)	13	1354.49 (793.70–2311.51)	2.64 (1.55–4.51)	15	1919.56 (1164.60–3163.94)	3.74 (2.27–6.17)	15	444.43 (266.94–739.93)	0.87 (.52–1.44)
**Part B**												
		Group-B3			Group-B10			Group-BPbo				
Day 1	18	689.36 (402.31–1181.24)	-	18	574.02 (385.58–854.56)	-	5	493.05 (92.19–2636.84)	-		-	
Day 15	18	2423.54 (1796.47–3269.50)	3.91 (2.90–5.27)	17	3596.47 (2644.67–4890.81)	5.80 (4.27–7.89)	5	589.81 (334.04–1041.42)	0.95 (.54–1.68)		-	
Day 31	17	2157.80 (1472.28–3162.53)	3.43 (2.34–5.03)	17	2847.07 (1944.62–4168.31)	4.53 (3.09–6.63)	5	602.35 (297.53–1219.45)	0.96 (.47–1.94)		-	
Month 6	18	1547.09 (1100.15–2175.61)	2.26 (1.61–3.17)	12	1677.07 (1103.28–2549.28)	2.45 (1.61–3.72)	5	540.16 (281.43–1036.75)	0.79 (.41–1.51)		-	

Abbreviations: CI, confidence interval; GMR, geometric mean ratio; GMT, geometric mean titer; *N*, number with pre and corresponding post results available; SARS-CoV-2, severe acute respiratory syndrome coronavirus 2.

At Day 1, the unadjusted GMT is calculated. At other time points, the adjusted GMT and GMR from baseline is calculated based on an analysis of covariance model on the log transformed titers with baseline log transformed titers as covariate, treatment group as fixed effects.

Baseline levels of neutralizing titers against the WT (D614G) variant were higher (around 2.7- to 4.5-fold) than those against the BA.5 variant (Supplementary Figure 2A and Supplementary Table 2). Nevertheless, increases in cross-neutralizing titers against the WT (D614G) were observed, with GMRs from baseline at Day 31 of 3.50 in Group-A10, 4.32 in Group-A30, and 6.91 in Group-A100 (Supplementary Table 2). There was a consistent dose-response observed at each time point with the highest responses observed in Group-A100. At Month 6, 45.5% of participants in Group-A10, 53.8% in Group-A30, and 60.0% in Group-A100 continued to have WT (D614G) cross-neutralizing titers at least 2-fold higher than at baseline (Supplementary Table 3).

### Part B

Among 88 individuals screened, 42 were enrolled and administered their assigned IP in Part B, and 40 (95.2%) completed the study (Supplementary Figure 2). Demographic and baseline characteristics of study groups are presented in Table 1. The median age of all participants at screening was 38.5 years (range 19–49), 50% (*n* = 21) were female, 93% (*n* = 39) were White, and 21% (*n* = 9) identified as Hispanic/Latino. All participants (100%) were N protein–antibody positive at Day 1.

### Safety

The overall frequency and intensity of solicited local and systemic AEs reported for Group-B10 appeared similar to those reported for Group-A10 and tended to be higher in Group-B3 (Table 2). Any solicited AE was reported for 77.8% of participants in Group-B3, 55.6% in Group-B10, versus 50.0% in Group-BPbo. The most frequently reported solicited AEs were injection site pain (50% in Group-B3, 33.3% in Group-B10, vs 16.7% in Group-BPbo), fatigue (38.9%, 27.8%, vs 16.7% in the respective groups), headache (33.3%, 22.2%, vs 33.3%, respectively) and myalgia (33.3%, 44.4%, vs 0%, respectively) (Figure 2*B*). No Grade 3 solicited AEs were reported in Part B. The median duration of solicited AEs was 1–2 days for a majority of AEs. The only AE with a median duration of 4 days or more was headache (Group-B3, 4 days).

The percentage of participants reporting any unsolicited AE was 44.4% in Group-B3, 11.1% in Group-B10, versus 16.7% in Group-BPbo (Table 2). None were of Grade 3 intensity. Unsolicited AEs considered causally related to the IP were prothrombin time prolonged (*n* = 3, Group-B3), rash (*n* = 1, Group-B3), joint stiffness (*n* = 1, Group-B3), and hypertension (*n* = 1, Group-B10). Other than the hypertension (Grade 2, unresolved at study end) in a participant with a medical history of “borderline high blood pressure,” all were Grade 1 in intensity and resolved.

Medically attended AEs until study end were reported by 3 participants (16.7%), all in Group-B3, and none were considered causally related to the IP (Table 2). There were no AESI, no SAEs, no deaths, and no study discontinuations due to an AE in Part B. No safety signals were observed from clinical safety laboratory test results or review of ECGs (not shown).

### Immunogenicity

Between 0 and 1 participant was excluded from the Day 15 and Day 31 per-protocol sets, and between 0 and 6 from the Month 6 per-protocol set, mainly due to missing endpoints or noncompliance with study procedures and assessments (Supplementary Figure 1B). All participants had evidence of prior SARS-CoV-2 infection, and levels of neutralizing titers against SARS-CoV-2 BA.5 prior to IP administration were high (Figure 3*B*). The percentage of participants with at least a doubling in neutralizing titers against the SARS-CoV-2 BA.5 variant at Day 15 was 72.2% for Group-B3% and 88.2% for Group-B10 (Table 3). A ≥ 4-fold change was observed in 38.9% and 76.5%, respectively at Day 15. At Day 31, the percentage of participants with a ≥ 4-fold change in neutralizing titers from baseline levels was 29.4% in Group-B3, and 64.7% in Group-B10, versus 0% in Group-BPbo. At Month 6, 44.4% of participants in Group-B3 and 58.3% in Group-B10 continued to have neutralizing titers at least 2-fold higher than at baseline.

Both doses of mRNA-CR-04 elicited neutralizing titers against SARS-CoV-2 BA.5, with numerically higher titers observed at each post-IP administration time point in Group-B10 (Table 4). At Day 31, the SARS-CoV-2 BA.5 neutralizing titer GMR from baseline was 3.43 in Group-B3, and 4.53 in Group-B10. These neutralizing titers had waned by Month 6 but remained above baseline titers in both mRNA-CR-04 groups.

As observed in Part A, baseline levels of neutralizing titers against the WT (D614G) variant were higher than those against the BA.5 variant (Supplementary Figure 2B and Supplementary Table 2). Nevertheless, increases in cross-neutralizing titers against the WT (D614G) were observed, with GMRs from baseline at Day 31 of 2.43 in Group-B3 and 3.98 in Group-B10 (Supplementary Table 2). The magnitude of the neutralizing titer response was higher in Group-B10 than Group-B3 at Days 15 and 31. At Month 6, 38.9% of participants in Group-B3 and 33.3% in Group-B10 continued to have WT (D614G) cross-neutralizing titers at least 2-fold higher than at baseline (Supplementary Table 3).

## DISCUSSION

This study evaluated a novel mRNA vaccine construct using the SARS-CoV-2 BA.5 S protein as a model antigen for evaluation in humans for the first time.

There was a trend toward a potential dose-related increase in reactogenicity in Part A; nevertheless, all mRNA-CR-04 doses were generally well tolerated. The type, frequency, and intensity of local and systemic solicited AEs were comparable to that reported for other authorized mRNA COVID-19 vaccine boosters [16, 17]. Injection site pain, fatigue, myalgia, and headache were the most frequently reported solicited AEs and were usually mild and transient. There were no safety concerns during the study, no discontinuations due to AEs, and no SAEs, medically attended AEs, or AESIs considered as causally related to the vaccine in either Part A or Part B.

As expected, there was a high rate of previous exposure to SARS-CoV-2 in study participants as evidenced by positive N protein serology during screening and relatively high baseline SARS-CoV-2 BA.5 neutralizing titers. All doses of mRNA-CR-04, including the lowest dose of 3 µg, elicited notable increases in SARS-CoV-2 BA.5 neutralizing titers and cross-neutralizing titers against the WT (D614G) variant. Neutralizing titers were declining by Month 6 but remained higher than baseline levels. There was a trend toward a higher magnitude of immune responses with increasing mRNA-CR-04 dose level.

A potential limitation of this Phase 1 study is the attrition of participants from the per-protocol set for the analysis of immunogenicity in some groups by Month 6. In view of the high immune responses observed, it is likely that the study conclusions would have been unchanged with a larger sample size at this time point.

In conclusion, the investigational mRNA-CR-04 vaccine induced the robust anticipated immune response against the encoded antigen in adult participants, and with no safety concerns deemed causally related to mRNA-CR-04 observed in doses ranging between 3 and 100 µg. Further investigation of potential vaccine candidates using this novel mRNA platform is warranted.

## Supplementary Material

ofaf689_Supplementary_Data

## Supplementary Data

Supplementary materials are available at *Open Forum Infectious Diseases* online. Consisting of data provided by the authors to benefit the reader, the posted materials are not copyedited and are the sole responsibility of the authors, so questions or comments should be addressed to the corresponding author.
