# Supplementary material for: Phase 1 Randomized Controlled Trial of the Safety and Immunogenicity of the SARS-CoV-2 (Omicron BA.5) mRNA-CR-04 Vaccine in Adults 18–49 Years of Age
Source: Open Forum Infect Dis. 2025 Nov 26;12(12):ofaf689. doi: 10.1093/ofid/ofaf689 (PMC12651558; doi:10.1093/ofid/ofaf689)
Supplement: ofaf689_Supplementary_Data [file ofaf689_supplementary_data.zip › Supplement_Revised version_clean.docx]

**Phase 1 randomized controlled trial of the safety and immunogenicity of the SARS-CoV-2 (Omicron BA.5) mRNA-CR-04 vaccine in adults 18–49 years of age**

Naficy Abdi, Venken Mireille, Xi Yingmei, Loughrey Mark, Maruggi Giulietta, Sharma Hema, Aggarwal Kunal, Brune Daniel, Nguyen Bach-Yen

#

# SUPPLEMENT

## Inclusion criteria

- Written or witnessed informed consent obtained from the participant prior to performance of any study-specific procedure.
- Participants, who in the opinion of the investigator, can and will comply with the requirements of the protocol (e.g., completion of the e-diary and study procedures).
- Has received 2 doses of primary series and booster dose(s) of an authorized or licensed mRNA coronavirus disease 2019 (COVID-19) vaccine (only Moderna or Pfizer vaccines) with the last booster dose administered at least 6 months or more prior to screening and has provided documentation of receiving the vaccination series (e.g., vaccination card).
- Negative for severe acute respiratory syndrome coronavirus 2 (SARS-CoV-2) infection by real-time polymerase chain reaction test at screening within 7 days prior to study vaccination.
- Is a male or nonpregnant female of 18 to 49 years, inclusive, at screening.
- If the participant is a woman of child-bearing age, the participant agrees to practice true abstinence or use at least one highly effective form of contraception for at least 30 days prior to study vaccination up to one month after study vaccination.
- Agrees to refrain from blood or plasma donation from screening and up to 6 months after vaccination.
- Is healthy or medically stable as determined by investigator judgment based on medical history, clinical laboratory tests, vital sign measurements, and physical examination findings.

## Exclusion criteria

- Has a new onset, clinically significant, abnormal biochemistry or hematology finding (defined as ≥ Grade 1) at screening (participants with Grade 1 laboratory abnormalities that have been stable for at least 6 months before enrollment may be included in the study).
- Has any medical disease or condition that, in the opinion of the investigator, precludes study participation. This includes any acute, subacute, intermittent, or chronic medical disease or condition that would place the participant at an unacceptable risk of injury, render the participant unable to meet the requirements of the protocol, or may interfere with the evaluation of responses or the participant’s successful completion of the trial.
- Any confirmed or suspected immunosuppressive or immunodeficient condition, based on medical history and physical examination (no laboratory testing required).
- History of myocarditis, pericarditis, second- and third-degree heart block or idiopathic cardiomyopathy, or presence of any medical condition that increases risk of myocarditis or pericarditis, including cocaine abuse, cardiomyopathy, endomyocardial fibrosis, hyper-eosinophilic syndrome, hypersensitivity myocarditis eosinophilic granulomatosis with polyangiitis, persistent myocardial viral infection (e.g., due to enterovirus or adenovirus).
- Has an acute febrile illness with a temperature ≥38.0°C or ≥100.4°F observed by the participant or at the study site within 72 hours prior to study vaccination. Participants with suspected COVID-19 symptoms should be excluded and referred for medical care.
- Has a history of hypersensitivity or severe allergic reaction, including anaphylaxis, generalized urticaria, angioedema, and other significant reactions to any previous vaccine, or any component of the study vaccine.
- Has a body mass index >40 kg/m².
- Has had known close contact with anyone who had a confirmed SARS-CoV-2 infection within 14 days before study vaccination.
- Has a history of documented SARS-CoV-2 infection or COVID-19 within 6 months before the date of screening visit.
- Has any self-reported or medically documented clinically significant medical or psychiatric condition. Significant medical conditions include, but are not limited to, the following:
  - Moderate or severe respiratory disease (e.g., chronic obstructive pulmonary disease, asthma).
  - Uncontrolled hypertension, defined as an average systolic blood pressure ≥140 mmHg or an average diastolic blood pressure ≥90 mmHg, based on an average of up to 3 blood pressure measurements.
  - Clinically significant cardiovascular disease (e.g., congestive heart failure, cardiomyopathy, ischemic heart disease).
  - Neurological or neurodevelopmental conditions (e.g., Down syndrome, dementia, chronic migraine not controlled by medication, epilepsy, stroke or seizures in the last 3 years, encephalopathy, focal neurologic deficits, Guillain Barré syndrome, encephalomyelitis, or transverse myelitis).
  - Ongoing malignancy or recent diagnosis of malignancy in the last 5 years (excluding basal cell and squamous cell carcinoma of the skin).
  - Tuberculosis or non-tuberculosis mycobacterial infection.
  - Autoimmune disease, including hypothyroidism without a defined nonautoimmune cause.
  - Immunodeficiency of any cause, including from solid organ transplant, blood, or bone marrow transplant, or use of other immune-weakening medicine.
  - Type 1 or 2 diabetes mellitus regardless of disease control.
- Has any of the following self-reported or medically documented risk factors for severe COVID-19
  - Chronic kidney disease
  - Cerebrovascular disease
  - Cystic fibrosis
  - Chronic liver disease
  - Pulmonary fibrosis
- Has participated or plans to participate in another investigational study involving any investigational drug or device within 60 days or 5 half-lives, whichever is longer, before study vaccination and throughout the study.
- Has received a licensed or authorized non-mRNA COVID-19 vaccine (primary series or booster dose).
- Has received or plans to receive any licensed vaccine within 4 weeks before or after study vaccination. Inactivated vaccines for influenza are permitted during the study if they are administered at least 14 days before or after study vaccination.
- Is planning to receive an authorized or licensed COVID-19 booster vaccination for the duration of the study (for participants who are not covered by local recommendations to receive booster per current standard of care) OR is planning to receive an authorized or licensed COVID-19 booster vaccination on or before Day 31 of the study (for participants covered by local recommendations to receive booster).
- Has received or plans to receive immunoglobulins or any blood or blood products within 90 days before study vaccination and throughout the study.
- Reports chronic use (more than 14 continuous days) of any medication that may be associated with changes in immune function including, but not limited to, systemic corticosteroids exceeding 20 mg/day of prednisone equivalent, allergy injections, immunoglobulins, interferons, immunomodulators, cytotoxic drugs, or other similar or toxic drugs within 6 months of study vaccination. Note: The use of low-dose topical, ophthalmic, inhaled, intra-articular, and intranasal steroid preparations is permitted.
- Pregnant or lactating female.
- Female participant planning to become pregnant or planning to discontinue contraceptive precautions within one month following study vaccination.
- Participant is an employee or family member of the investigator or study site personnel.

## Study hold rules

| **Holding Rule** | **Event** | **Number or percentage of participants to pause vaccination in all groups, pending further evaluation by the iSRC** |
| --- | --- | --- |
| 1a | Death or any life-threatening SAE regardless of causality | ≥1 |
| 1b | Any non-life-threatening SAE that cannot be reasonably attributed to a cause other than vaccination as per investigator or sponsor assessment | ≥1 |
| 1c | Any withdrawal from the study (by investigator or participant request) following a Grade 3 AE | ≥1 |
| 1d | Any administration site or systemic solicited AE leading to hospitalization,  OR  necrosis at the injection site,  each with an event onset within the 7-day (Day 1–7) post-vaccination period | ≥1 |
| 2a* | Any Grade 3 solicited administration site events, with an event onset within the 7-day (Day 1–7) post-vaccination period | ≥ 3/6 or 30%** |
| 2b* | Any Grade 3 solicited systemic events, with an event onset within the 7-day (Day 1–7) post-vaccination period | ≥ 3/6 or 30%** |
| 2c* | Any Grade 3 unsolicited AE, that can be reasonably attributed to the vaccination as per investigator or sponsor assessment, with an event onset within the 7-day (Day 1–7) post-vaccination period  OR  any Grade 3 or above abnormality in pre-specified hematological or biochemical laboratory parameters with an event onset within the 8-day (Day 1–8) post-vaccination period except for non-clinically significant leukopenia | ≥ 2/6 or 10%*** |

AE: adverse event; iSRC: internal safety review committee; SAE: serious adverse event

*Refer to the United States Food and Drug Administration guidance for industry “Toxicity grading scale for healthy adults and adolescent volunteers enrolled in preventive vaccine clinical trials”.^1^

**3/6 sentinel participants or 30% of all participants, whichever is met first

***2/6 sentinel participants or 10% of all participants, whichever is met first

**Determination of AE intensity**

The investigator will make an assessment of intensity for each adverse event (AE), adverse event of special interest (AESI), and serious adverse event (SAE) reported during the study and assign it to one of the following categories:

Mild: A type of AE that is usually transient and may require only minimal treatment or therapeutic intervention. The event does not generally interfere with usual activities of daily living.

Moderate: A type of AE that is usually alleviated with additional specific therapeutic intervention. The event interferes with usual activities of daily living, causing discomfort but poses no significant or permanent risk of harm to the research participant.

Severe: A type of AE that interrupts usual activities of daily living, or significantly affects clinical status, or may require intensive therapeutic intervention.

The intensity of the solicited AEs will be assessed as described below.

# SUPPLEMENTARY TABLES

**Table S1** Intensity scale for solicited events

| **Event** | **Intensity grade** | **Parameter** |
| --- | --- | --- |
| Pain at administration site | 0 | None |
|  | 1 | Mild: Any pain neither interfering with nor preventing normal everyday activities |
|  | 2 | Moderate: Painful when limb is moved and interferes with everyday activities |
|  | 3 | Severe: Significant pain at rest, prevents normal everyday activities |
| Redness at administration site | 0 | Greatest surface diameter < 25 mm |
|  | 1 | Greatest surface diameter 25–50 mm |
|  | 2 | Greatest surface diameter 51–100 mm |
|  | 3 | Greatest surface diameter > 100 mm |
| Swelling at administration site | 0 | Greatest surface diameter < 25 mm |
|  | 1 | Greatest surface diameter 25–50 mm |
|  | 2 | Greatest surface diameter 51–100 mm |
|  | 3 | Greatest surface diameter > 100 mm |
| Temperature | 0 | < 38.0°C (100.4°F) |
|  | 1 | 38.0°C (100.4°F) to 38.4°C (101.1°F) |
|  | 2 | 38.5°C (101.2°F) to 38.9°C (102.0°F) |
|  | 3 | >38.9°C (102.0°F) |
| Arthralgia (joint pain) | 0 | None |
|  | 1 | Mild: Arthralgia present but does not interfere with activity |
|  | 2 | Moderate: Arthralgia that interferes with normal activity |
|  | 3 | Severe: Arthralgia that prevents normal activity |
| Headache | 0 | None |
|  | 1 | Mild: Headache that is easily tolerated |
|  | 2 | Moderate: Headache that interferes with normal activity |
|  | 3 | Severe: Headache that prevents normal activity |
| Fatigue (tiredness) | 0 | None |
|  | 1 | Mild: Fatigue that is easily tolerated |
|  | 2 | Moderate: Fatigue that interferes with normal activity |
|  | 3 | Severe: Fatigue that prevents normal activity |
| Lymphadenopathy | 0 | None |
|  | 1 | Mild: No interference with activity |
|  | 2 | Moderate: Some interference with daily activity or requires repeated use of non-narcotic pain reliever |
|  | 3 | Severe: Prevents daily activity or requires use of narcotic pain reliever |
| Chills | 0 | None |
|  | 1 | Mild sensation of cold; shivering; chattering of teeth |
|  | 2 | Moderate tremor of the entire body; narcotics indicated |
|  | 3 | Severe or prolonged, not responsive to narcotics |
| Myalgia (muscle pain) | 0 | None |
|  | 1 | Mild: Myalgia present but does not interfere with activity |
|  | 2 | Moderate: Myalgia that interferes with normal activity |
|  | 3 | Severe: Myalgia that prevents normal activity |
| Abdominal pain | 0 | Normal |
|  | 1 | Mild: No interference with daily activity |
|  | 2 | Moderate: Interferes with daily activity |
|  | 3 | Severe: Prevents daily activity |
| Vomiting | 0 | Normal |
|  | 1 | Mild: No interference with daily activity or 1–2 episodes in 24 hours |
|  | 2 | Some interference with activity or >2 episodes in 24 hours |
|  | 3 | Severe: Prevents daily activity, requires outpatient intravenous hydration |
| Diarrhea | 0 | Normal |
|  | 1 | Mild: 2-3 loose stools or <400g in 24 hours |
|  | 2 | Moderate: 4-5 stools or 400-800 g in 24 hours |
|  | 3 | Severe: 6 or more water stools or >800g in 24 hours  or requires outpatient intravenous hydration |

The toxicity grading scale for clinical laboratory test results was derived from the FDA guidance for industry: toxicity grading scale for healthy adult and adolescent volunteers enrolled in preventive vaccine clinical trials.^1^

**Table S2** Adjusted SARS-CoV-2 wild type (D614G) neutralizing geometric mean titers and geometric mean ratios from baseline (per protocol set)

| **Time point** | **Group-A10** | | | **Group-A30** | | | **Group-A100** | | | | **Group-APbo** | | |
| --- | --- | --- | --- | --- | --- | --- | --- | --- | --- | --- | --- | --- | --- |
|  | **N** | **GMT**  **(95% CI)** | **GMR**  **(95% CI)** | **N** | **GMT**  **(95% CI)** | **GMR**  **(95% CI)** | **N** | **GMT**  **(95% CI)** | **GMR**  **(95% CI)** | **N** | | **GMT**  **(95% CI)** | **GMR**  **(95% CI)** |
| **Part A** |  |  |  |  |  |  |  |  |  |  | |  |  |
| Day 1 | 16 | 2502.06  (1839.63; 3403.03) | - | 17 | 1409.21  (931.99; 2130.80) | - | 17 | 1820.75  (1080.88; 3067.05) | - | 18 | | 1421.87  (706.04; 2863.44) | - |
| Day 15 | 16 | 8073.12  (5651.68; 11532.03) | 4.68  (3.28; 6.69) | 17 | 9990.27  (7096.52; 14064.02) | 5.80  (4.12; 8.16) | 17 | 14543.96  (10348.31; 20440.70) | 8.44  (6.00; 11.86) | 18 | | 1654.35  (1186.60; 2306.48) | 0.96  (0.69; 1.34) |
| Day 31 | 14 | 6251.22  (4450.68; 8780.17) | 3.50  (2.49; 4.91) | 17 | 7726.20  (5712.62; 10449.51) | 4.32  (3.20; 5.85) | 16 | 12351.27  (9068.09; 16823.16) | 6.91  (5.07; 9.41) | 18 | | 1585.37  (1182.26; 2125.92) | 0.89  (0.66; 1.19) |
| Month 6 | 11 | 3813.02  (2511.62; 5788.74) | 1.99  (1.31; 3.03) | 13 | 3970.88  (2713.92; 5810.01) | 2.08  (1.42; 3.04) | 15 | 4645.04  (3267.21; 6603.92) | 2.43  (1.71; 3.45) | 15 | | 2441.97  (1715.51; 3476.06) | 1.28  (0.90; 1.82) |
| **Part B** |  |  |  |  |  |  |  |  |  |  | |  |  |
|  |  | **Group-B3** |  |  | **Group-B10** |  |  | **Group-BPbo** |  |  | |  |  |
| Day 1 | 18 | 2139.66  (1353.70; 3381.96) | - | 18 | 1604.51  (1013.28; 2540.73) | - | 5 | 1702.33  (571.67; 5069.18) | - |  | | - |  |
| Day 15 | 18 | 4661.13  (3673.40; 5914.45) | 2.53  (1.99; 3.21) | 17 | 6363.85  (4982.55; 8128.08) | 3.45  (2.70; 4.41) | 5 | 2129.10  (1358.56; 3336.68) | 1.15  (0.74; 1.81) |  | | - |  |
| Day 31 | 17 | 4633.14  (3382.40; 6346.37) | 2.43  (1.77; 3.32) | 17 | 7601.57  (5553.99; 10404.02) | 3.98  (2.91; 5.45) | 5 | 2819.98  (1583.73; 5021.26) | 1.48  (0.83; 2.63) |  | | - |  |
| Month 6 | 18 | 4059.06  (3244.67; 5077.85) | 2.01  (1.61; 2.51) | 12 | 3342.96  (2541.75; 4396.73) | 1.65  (1.26; 2.18) | 5 | 2571.87  (1680.53; 3935.97) | 1.27  (0.83; 1.95) |  | | - |  |

CI: confidence interval; GMR: geometric mean ratio; GMT: geometric mean titer; N: number with pre and corresponding post results available; SARS-CoV-2: severe acute respiratory syndrome coronavirus 2

At Day 1, the unadjusted GMT is calculated. At other time points, the adjusted GMT and GMR from baseline is calculated based on an analysis of covariance model on the log transformed titers with baseline log transformed titers as covariate, treatment group as fixed effects.

**Table S3** Fold change from baseline in unadjusted SARS-CoV-2 wild type (D614G) neutralizing titers (per protocol set)

|  |  | **Group-A10** | | | **Group-A30** | | | **Group-A100** | | | **Group-APbo** | | |
| --- | --- | --- | --- | --- | --- | --- | --- | --- | --- | --- | --- | --- | --- |
| **Time point** | **Fold change** | **N** | **n** | **% (95% CI)** | **N** | **n** | **% (95% CI)** | **N** | **n** | **% (95% CI)** | **N** | **n** | **% (95% CI)** |
| **Part A** | | | | |  |  |  |  |  |  |  |  |  |
| Day 15 | < 2 | 16 | 2 | 12.5 (1.6–38.3) | 17 | 2 | 11.8 (1.5–36.4) | 17 | 0 | 0.0 (0.0–19.5) | 18 | 18 | 100 (81.5–100) |
|  | ≥ 2 | 16 | 14 | 87.5 (61.7–98.4) | 17 | 15 | 88.2 (63.6–98.5) | 17 | 17 | 100 (80.5–100) | 18 | 0 | 0.0 (0.0–18.5) |
|  | ≥ 4 | 16 | 6 | 37.5 (15.2–64.6) | 17 | 8 | 47.1 (23.0–72.2) | 17 | 12 | 70.6 (44.0–89.7) | 18 | 0 | 0.0 (0.0–18.5) |
|  | ≥ 8 | 16 | 3 | 18.8 (4.0–45.6) | 17 | 6 | 35.3 (14.2–61.7) | 17 | 9 | 52.9 (27.8–77.0) | 18 | 0 | 0.0 (0.0–18.5) |
| Day 31 | < 2 | 14 | 4 | 28.6 (8.4–58.1) | 17 | 3 | 17.6 (3.8–43.4) | 16 | 0 | 0.0 (0.0–20.6) | 18 | 18 | 100 (81.5–100) |
|  | ≥ 2 | 14 | 10 | 71.4 (41.9–91.6) | 17 | 14 | 82.4 (56.6–96.2) | 16 | 16 | 100 (79.4–100) | 18 | 0 | 0.0 (0.0–18.5) |
|  | ≥ 4 | 14 | 5 | 35.7 (12.8–64.9) | 17 | 8 | 47.1 (23.0–72.2) | 16 | 11 | 68.8 (41.3–89.0) | 18 | 0 | 0.0 (0.0–18.5) |
|  | ≥ 8 | 14 | 1 | 7.1 (0.2–33.9) | 17 | 5 | 29.4 (10.3–56) | 16 | 7 | 43.8 (19.8–70.1) | 18 | 0 | 0.0 (0.0–18.5) |
| Month 6 | < 2 | 11 | 6 | 54.5 (23.4–83.3) | 13 | 6 | 46.2 (19.2–74.9) | 15 | 6 | 40.0 (16.3–67.7) | 15 | 11 | 73.3 (44.9–92.2) |
|  | ≥ 2 | 11 | 5 | 45.5 (16.7–76.6) | 13 | 7 | 53.8 (25.1–80.8) | 15 | 9 | 60.0 (32.3–83.7) | 15 | 4 | 26.7 (7.8–55.1) |
|  | ≥ 4 | 11 | 2 | 18.2 (2.3–51.8) | 13 | 3 | 23.1 (5.0–53.8) | 15 | 4 | 26.7 (7.8–55.1) | 15 | 2 | 13.3 (1.7–40.5) |
|  | ≥ 8 | 11 | 0 | 0.0 (0.0–28.5) | 13 | 1 | 7.7 (0.2–36.0) | 15 | 0 | 0.0 (0.0–21.8) | 15 | 0 | 0.0 (0.0–21.8) |
| **Part B** | | | | |  |  |  |  |  |  |  |  |  |
|  |  |  |  | **Group-B3** |  |  | **Group-B10** |  |  | **Group-BPbo** |  |  |  |
| Day 15 | < 2 | 18 | 7 | 38.9 (17.3–64.3) | 17 | 4 | 23.5 (6.8–49.9) | 5 | 5 | 100 (47.8–100) |  |  | - |
|  | ≥ 2 | 18 | 11 | 61.1 (35.7–82.7) | 17 | 13 | 76.5 (50.1–93.2) | 5 | 0 | 0.0 (0.0–52.2) |  |  | - |
|  | ≥ 4 | 18 | 3 | 16.7 (3.6–41.4) | 17 | 9 | 52.9 (27.8–77.0) | 5 | 0 | 0.0 (0.0–52.2) |  |  | - |
|  | ≥ 8 | 18 | 0 | 0.0 (0.0–18.5) | 17 | 3 | 17.6 (3.8–43.4) | 5 | 0 | 0.0 (0.0–52.2) |  |  | - |
| Day 31 | < 2 | 17 | 8 | 47.1 (23.0–72.2) | 17 | 3 | 17.6 (3.8–43.4) | 5 | 4 | 80.0 (28.4–99.5) |  |  | - |
|  | ≥ 2 | 17 | 9 | 52.9 (27.8–77.0) | 17 | 14 | 82.4 (56.6–96.2) | 5 | 1 | 20 (0.5–71.6) |  |  | - |
|  | ≥ 4 | 17 | 4 | 23.5 (6.8–49.9) | 17 | 8 | 47.1 (23-72.2) | 5 | 0 | 0.0 (0.0–52.2) |  |  | - |
|  | ≥ 8 | 17 | 0 | 0.0 (0.0–19.5) | 17 | 4 | 23.5 (6.8–49.9) | 5 | 0 | 0.0 (0.0–52.2) |  |  | - |
| Month 6 | < 2 | 18 | 11 | 61.1 (35.7–82.7) | 12 | 8 | 66.7 (34.9–90.1) | 5 | 4 | 80.0 (28.4–99.5) |  |  | - |
|  | ≥ 2 | 18 | 7 | 38.9 (17.3–64.3) | 12 | 4 | 33.3 (9.9–65.1) | 5 | 1 | 20.0 (0.5–71.6) |  |  | - |
|  | ≥ 4 | 18 | 2 | 11.1 (1.4–34.7) | 12 | 3 | 25.0 (5.5–57.2) | 5 | 0 | 0.0 (0.0–52.2) |  |  | - |
|  | ≥ 8 | 18 | 0 | 0.0 (0.0–18.5) | 12 | 0 | 0.0 (0.0–26.5) | 5 | 0 | 0.0 (0.0–52.2) |  |  | - |

CI: confidence interval; N: number with pre and corresponding post results available; n (%): number (percentage) with the specified fold change; SARS-CoV-2: severe acute respiratory syndrome coronavirus 2

# SUPPLEMENTARY FIGURES

**Figure S1** Part A and Part B – Participant flow

**ALT TEXT:** Graphical representation of the flow of participants through the study Parts A and B showing the number in each group, and the number excluded from different analysis sets with reasons for their exclusion.


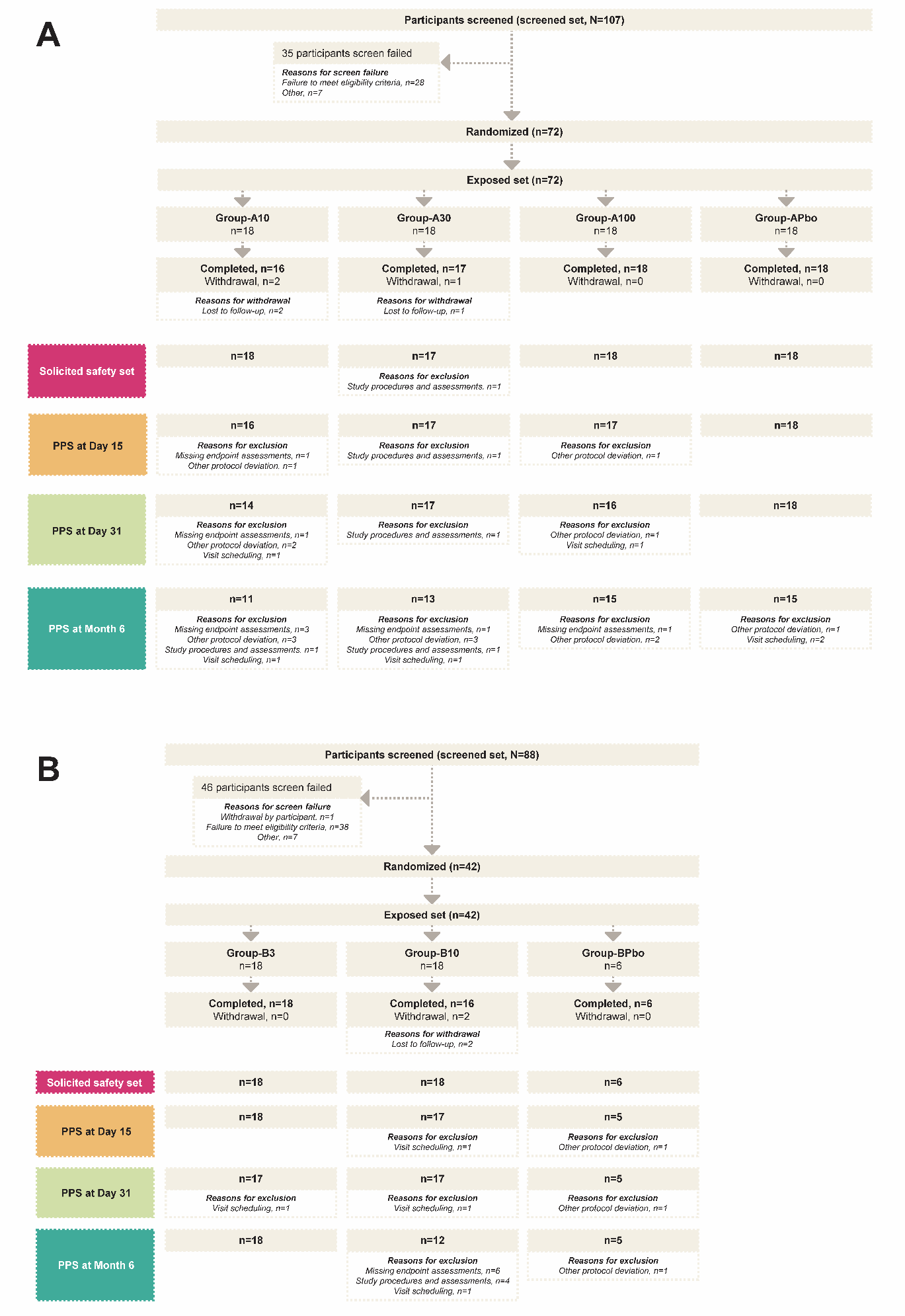


N: number of participants; n: number of participants in a given category; PPS: per protocol set

Counts of reasons for exclusion may not equal the number of participants excluded as some participants had multiple reasons.

Protocol deviations that could lead from elimination from the per protocol analysis set included:

- Receipt of any prohibited medication or vaccine
- Any intercurrent medical condition that could alter the participant’s immune response or confirmed to have an alteration of the participant’s initial immune status
- Major protocol deviation linked to the study eligibility criteria
- Study intervention not administered as specified by the protocol
- Failure to comply with the post-dose immunogenicity blood sampling schedule at a given timepoint

**Figure S2** Unadjusted geometric mean titers for neutralizing titers against SARS-CoV-2 wild type (D614G) by timepoint: (A) Part A, (B) Part B (per protocol set)

**ALT TEXT:** Graph showing the immune response in each study group against the SARS-CoV-2 wild type strain from before the first dose until 6 months after investigational product administration. The graphs use a logarithmic scale allowing an overview of the percentage change in immune response over time.


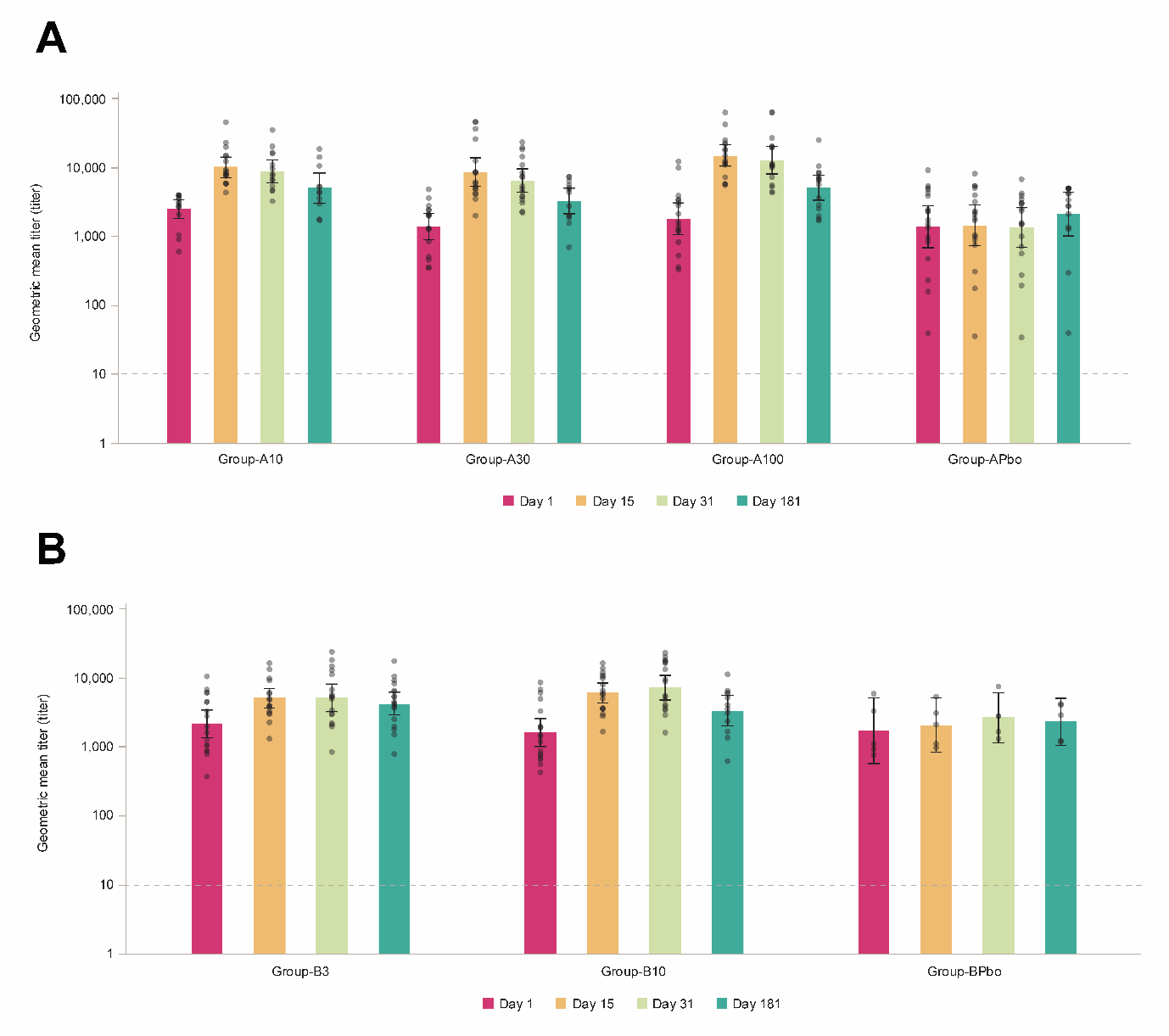


GMT: geometric mean titers; SARS-CoV-2: severe acute respiratory syndrome coronavirus 2

Circles represent individual datapoints, horizontal line represents the assay lower limit of quantitation, whiskers indicate 95% confidence intervals.

# Supplementary reference

1**.** Center for Biologics Evaluation and Research (CBER). Guidance for Industry Toxicity: Grading Scale for Healthy Adult and Adolescent Volunteers Enrolled in Preventive Vaccine Clinical Trials. Food and Drug Administration (FDA); 2007. Available at <https://www.fda.gov/media/73679/download>. Accessed 26 September 2025.
